# Supplementary material for: Bacterial Communities Related to Aroma Formation during Spontaneous Fermentation of ‘Cabernet Sauvignon’ Wine in Ningxia, China
Source: Foods. 2022 Sep 9;11(18):2775. doi: 10.3390/foods11182775 (PMC9497756; doi:10.3390/foods11182775)
Supplement: Supplementary file 1 [file foods-11-02775-s001.zip › foods-1891407-supplementary.pdf]

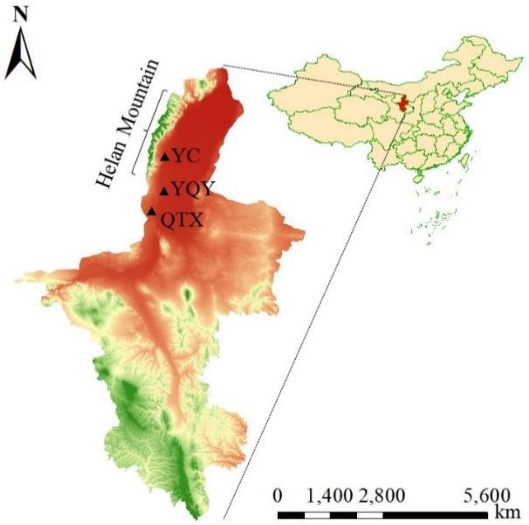

Figure S1. The geographical location of vineyards in the three sub-regions where grapes were obtained: YC, Yinchuan sub-region; YQY, Yuquanying sub-region; QTX, Qingtongxia sub-region.

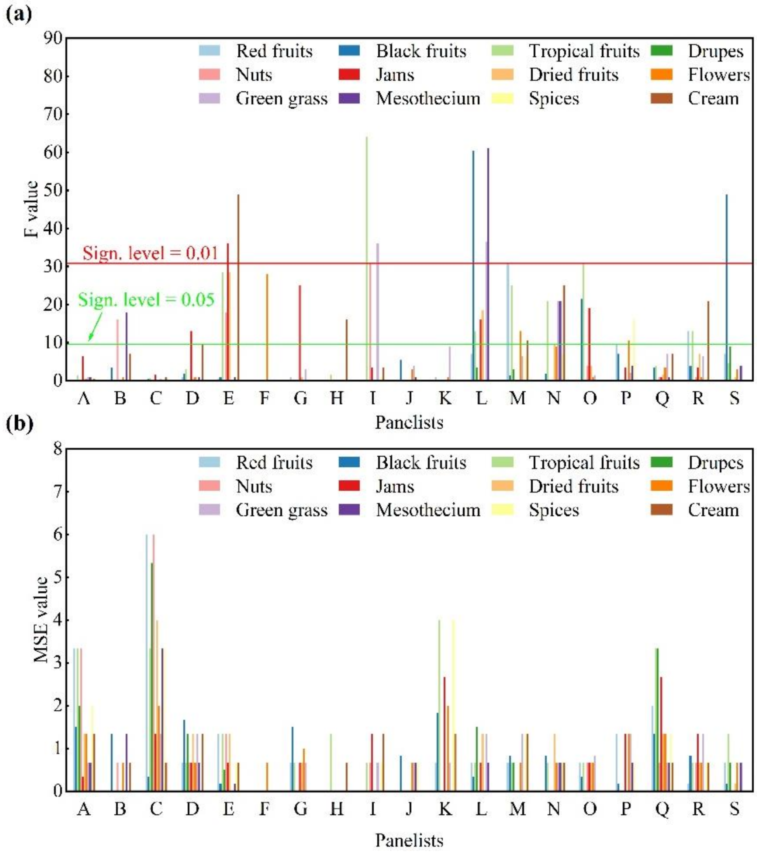

Figure S2. Accuracy and repeatability of 19 panelists. (a) The F value is used to assess the accuracy of each panelist's evaluation for each aroma descriptor, where a higher value indicates a better accuracy. Among the 19 panelists, 5 of them (A#, C#, J#, K#, and Q#) could not accurately recognize any of the aroma categories. (b) The MSE value is utilized to evaluate the repeatability of each panelist during the two tasting sessions, where a lower value illustrates better repeatability. Among the 19 panelists, 4 of them (A#, C#, K#, and Q#) had weak reproducibility in two consecutive tastings. Therefore, 14 panelists (B#, D#, E#, F#, G#, H#, I#, L#, M#, N#, O#, P#, R#, and S#) were chosen to

participate in the formal sensory evaluation for the spontaneously fermented wines.

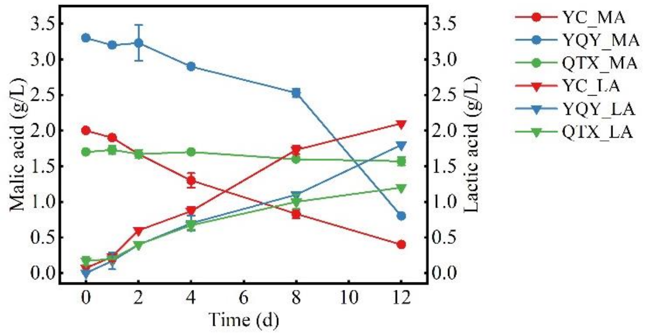

Figure S3: Monitoring the malolactic fermentation process. Malic and lactic acid were determined by Fourier-transform mid-infrared spectroscopy (FOSS WineScan, Hillerød, Denmark). YC_MA: The concentration of malic acid in Yinchuan samples; YC_LA: The concentration of lactic acid in Yinchuan samples. The same as YQY_MA, QTX_MA, YQY_LA, and QTX_LA.

Table S1. VOCs measured in the HS-SPME-GC-MS method, their retention indices (RIs), calculated retention indices (CRIs), and extracted ions for peak area calculation

Categories	VOCs	RI	CRI	Extracted ion (*m*/*z*)
Acetic esters	Isobutyl acetate	1002	1001	43
	Butyl acetate	1072	1075	43
	Isoamyl acetate	1121	1121	43
	Hexyl acetate	1275	1273	43
	cis-3-Hexenyl acetate	1300	1302	43
	trans -3-Hexenyl acetate	1308	1310	43
	Benzyl acetate	1710	1711	108
	2-Phenylethyl acetate	1801	1799	104
Methyl esters	Methyl octanoate	1388	1390	74
Ethyl esters	Ethyl butyrate	1026	1025	71
	Ethyl 2-methylbutyrate	1050	1051	102
	Ethyl 3-methylbutyrate	1067	1066	88
	Ethyl hexanoate	1232	1232	88
	Ethyl heptanoate	1336	1335	88
	Ethyl lactate	1340	1342	45
	Ethyl octanoate	1440	1438	88
	Ethyl nonanoate	1535	1533	88
	Ethyl decanoate	1662	1657	88
	Diethyl succinate	1675	1675	101
	Ethyl 9-decenoate	1688	1688	88
	Ethyl undecanoate	1739	1741	88
	Ethyl 2-phenylacetate	1780	1775	91
	Ethyl dodecanoate	1850	1844	88
	Ethyl tetradecanoate	2040	2034	88
	Ethyl hexadecanoate	2270	2270	88

Isobutyl esters	Isobutyl hexanoate	1356	1350	99
	Isobutyl decanoate	1750	1749	155
Isoamyl esters	Isoamyl hexanoate	1450	1452	70
	Isoamyl lactate	1570	1564	45
	Isoamyl octanoate	1657	1661	70
	Isoamyl decanoate	1868	1864	70
Straight chain fatty alcohols	1-Propanol	1038	1040	59
	1-Butanol	1148	1150	56
	1-Octanol	1559	1560	56
	1-Nonanol	1665	1666	56
	1-Decanol	1767	1769	70
Branched chain fatty alcohols	2-Methyl-1-propanol	1094	1096	43
	3-Methyl-1-butanol	1202	1200	55
	4-Methyl-1-pentanol	1316	1319	56
	2-Heptanol	1321	1322	45
	3-Methyl-1-pentanol	1330	1330	56
	3-Octenol	1451	1450	57
	2-Nonanol	1521	1521	45
	2,6,8-trimethyl-4-Nonanol	-	1568	69
	2,3-Butanediol	1583	1583	45
	cis-3-Nonenol	1682	1680	68
	trans-6-Nonenol	1714	1715	67
C6 alcohols	1-Hexanol	1359	1359	56
	cis -3-Hexenol	1386	1385	67
	trans-2-Hexenol	1394	1399	57
	cis -2-Hexenol	1417	1416	57
Aromatic alcohols	Benzyl alcohol	1866	1866	108
	2-Phenylethanol	1899	1899	91
Ketones	2,3-Pentanedione	1054	1057	43
	Acetoin	1278	1278	45
	2-Nonanone	1387	1389	58
	2,6,8-trimethyl-4-Nonanone	-	1398	57
	2-Undecanone	1580	1585	43
Fatty aldehydes	Hexanal	1080	1080	56
	Nonanal	1397	1393	57
	Decanal	1494	1494	57
Aromatic aldehydes	Benzaldehyde	1508	1508	106
	Phenylacetaldehyde	1638	1648	91
Fatty acids	Acetic acid	1452	1445	43
	Octanoic acid	2038	2042	60
	Nonanoic acid	2144	2150	60
	Decanoic acid	2279	2279	73
Terpenes	Linalool	1548	1548	71

	Citronellol	1767	1772	69
Aromatic alkenes	Styrene	1250	1251	104
Sulfides	Methionol	1710	1710	106
Volatile phenols	2,4-Di-tert-butylphenol	2330	2326	191

Note: The RIs on the DB-Wax column were obtained from the NIST Chemistry WebBook (https://webbook.nist.gov/). The CRIs were calculated according to the retention times of C8–C40 n-alkanes and each VOC.

Table S2. Oenological parameters of spontaneously fermented wines

Physicochemical indices	Sub-regions		
	YC	YQY	QTX
Residual sugar/g·L^{-1}	3.80 ± 0.04 ^{a}	2.52 ± 0.04 ^{c}	3.10 ± 0.03 ^{b}
Specific gravity	0.995 ± 0.000 ^{ab}	0.994 ± 0.000 ^{ab}	0.996 ± 0.000 ^{a}
Alcohol (%, v/v)	15.00 ± 0.08 ^{a}	13.30 ± 0.05 ^{c}	13.90 ± 0.02 ^{b}
Titratable acid/g·L^{-1}	6.51 ± 0.04 ^{b}	5.80 ± 0.04 ^{c}	7.02 ± 0.04 ^{a}
Volatile acid/g·L^{-1}	0.54 ± 0.01 ^{a}	0.39 ± 0.02 ^{b}	0.34 ± 0.01 ^{c}
pH	3.89 ± 0.00 ^{ab}	3.71 ± 0.00 ^{bc}	3.65 ± 0.00 ^{c}
Glycerol/g·L^{-1}	10.10 ± 0.10 ^{a}	9.60 ± 0.00 ^{c}	9.90 ± 0.00 ^{b}

Note: Different Latin letters indicate significant differences according to Duncan's test ($p < 0.05$). Residual sugar, alcohol, titratable acid, and volatile acid were measured according to the National Standard of China (GB/T 15038-2006, 2006); Specific gravity, pH, and glycerol were determined through Fourier-transform mid-infrared spectroscopy (FOSS WineScan, Hillerød, Denmark).

Table S3. The relative concentration of VOCs in different stages of spontaneous fermentation

VOCs	Sub-regions	Relative concentration (%)					
		A (0 d)	B (1 d)	C (2 d)	D (4 d)	E (8 d)	F (12 d)
Acetic esters	YC	n.d.	0.44 ± 0.05 ^{e}	7.96 ± 0.22 ^{b}	23.79 ± 0.77 ^{a}	5.28 ± 0.20 ^{c}	3.20 ± 0.32 ^{d}
	YQY	3.23 ± 2.06 ^{d}	7.21 ± 1.72 ^{c}	36.00 ± 3.39 ^{a}	14.45 ± 1.21 ^{b}	5.35 ± 1.53 ^{cd}	2.85 ± 0.84 ^{d}
	QTX	0.03 ± 0.01 ^{f}	0.61 ± 0.05 ^{e}	2.80 ± 0.42 ^{b}	4.60 ± 0.21 ^{a}	1.62 ± 0.09 ^{c}	1.19 ± 0.00 ^{d}
Methyl esters	YC	n.d.	0.03 ± 0.01 ^{bc}	0.02 ± 0.00 ^{d}	0.05 ± 0.00 ^{a}	0.03 ± 0.00 ^{b}	0.03 ± 0.01 ^{c}
	YQY	n.d.	0.01 ± 0.00 ^{b}	0.01 ± 0.00 ^{b}	0.05 ± 0.00 ^{a}	0.05 ± 0.00 ^{a}	0.05 ± 0.00 ^{a}
	QTX	n.d.	0.03 ± 0.00 ^{c}	0.03 ± 0.00 ^{d}	0.06 ± 0.01 ^{a}	0.05 ± 0.00 ^{ab}	0.05 ± 0.00 ^{b}
Ethyl esters	YC	0.09 ± 0.01 ^{c}	0.39 ± 0.03 ^{c}	0.45 ± 0.02 ^{c}	19.11 ± 0.27 ^{b}	24.91 ± 1.25 ^{a}	19.07 ± 2.70 ^{b}
	YQY	0.11 ± 0.01 ^{d}	0.66 ± 0.38 ^{d}	1.12 ± 0.07 ^{d}	37.70 ± 0.63 ^{c}	52.82 ± 2.83 ^{a}	44.27 ± 1.21 ^{b}
	QTX	0.13 ± 0.01 ^{c}	0.31 ± 0.02 ^{c}	0.58 ± 0.09 ^{c}	40.04 ± 1.46 ^{b}	44.17 ± 3.62 ^{a}	40.32 ± 2.42 ^{b}
Isobutyl esters	YC	n.d.	n.d.	n.d.	0.01 ± 0.00 ^{b}	0.01 ± 0.00 ^{a}	0.01 ± 0.00 ^{c}
	YQY	n.d.	n.d.	n.d.	0.05 ± 0.00 ^{a}	0.06 ± 0.01 ^{a}	0.04 ± 0.00 ^{b}
	QTX	n.d.	n.d.	n.d.	0.02 ± 0.00 ^{a}	0.02 ± 0.00 ^{a}	0.02 ± 0.00 ^{b}
Isoamyl esters	YC	0.10 ± 0.02 ^{d}	0.42 ± 0.03 ^{a}	0.39 ± 0.04 ^{ab}	0.18 ± 0.02 ^{c}	0.40 ± 0.05 ^{a}	0.33 ± 0.05 ^{b}
	YQY	n.d.	n.d.	n.d.	0.36 ± 0.01 ^{c}	1.01 ± 0.06 ^{a}	0.66 ± 0.03 ^{b}
	QTX	0.10 ± 0.01 ^{e}	0.40 ± 0.01 ^{d}	0.39 ± 0.01 ^{d}	0.72 ± 0.01 ^{c}	1.21 ± 0.10 ^{a}	1.08 ± 0.05 ^{b}
Straight chain fatty alcohols	YC	0.25 ± 0.01 ^{c}	6.54 ± 0.50 ^{a}	3.18 ± 0.14 ^{b}	0.41 ± 0.03 ^{c}	0.18 ± 0.03 ^{c}	0.18 ± 0.01 ^{c}
	YQY	0.31 ± 0.03 ^{b}	1.59 ± 0.07 ^{a}	1.70 ± 0.15 ^{a}	0.30 ± 0.01 ^{b}	0.11 ± 0.01 ^{c}	0.16 ± 0.01 ^{c}
	QTX	0.33 ± 0.02 ^{b}	2.88 ± 0.22 ^{a}	3.05 ± 0.21 ^{a}	0.18 ± 0.18 ^{b}	0.17 ± 0.01 ^{b}	0.18 ± 0.02 ^{b}
	YC	1.54 ± 0.19 ^{c}	7.14 ± 0.25 ^{b}	8.00 ± 0.29 ^{b}	14.84 ± 1.18 ^{a}	16.91 ± 2.62 ^{a}	16.44 ± 2.39 ^{a}

Branched chain fatty alcohols	YQY	2.07 ± 0.30 ^{b}	11.43 ± 0.53 ^{a}	11.50 ± 0.85 ^{a}	13.47 ± 1.46 ^{a}	11.46 ±1.89 ^{a}	12.35 ± 2.28 ^{a}
	QTX	2.88 ± 0.70 ^{d}	5.30 ± 0.42 ^{cd}	10.27 ± 0.59 ^{ab}	12.08 ± 0.94 ^{ab}	7.73 ± 5.93 ^{bc}	14.21 ± 1.30 ^{a}
C6 alcohols	YC	67.50 ± 4.47 ^{b}	73.65 ± 0.56 ^{a}	47.07 ± 1.75 ^{c}	4.03 ± 0.08 ^{d}	1.24 ± 0.11 ^{d}	0.85 ± 0.12 ^{d}
	YQY	63.54 ± 0.89 ^{a}	65.17 ± 1.54 ^{a}	31.04 ± 2.07 ^{b}	2.58 ± 0.17 ^{c}	0.48 ± 0.02 ^{d}	0.76 ± 0.13 ^{cd}
	QTX	63.46 ± 2.53 ^{b}	81.35 ± 0.12 ^{a}	62.97 ± 2.00 ^{b}	1.35 ± 0.11 ^{c}	0.69 ± 0.03 ^{c}	0.83 ± 0.12 ^{c}
Aromatic alcohols	YC	0.32 ± 0.02 ^{d}	1.73 ± 0.26 ^{d}	3.33 ± 0.24 ^{d}	21.74 ±2.18 ^{c}	47.09 ± 2.93 ^{b}	56.92 ± 5.19 ^{a}
	YQY	0.53 ± 0.07 ^{d}	6.79 ± 0.60 ^{c}	10.39 ± 2.00 ^{c}	21.09 ± 2.92 ^{b}	25.87 ± 5.46 ^{b}	35.82 ± 2.35 ^{a}
	QTX	0.46 ± 0.03 ^{c}	1.72 ± 0.05 ^{c}	2.99 ± 0.15 ^{c}	36.60 ± 2.63 ^{b}	42.46 ± 2.44 ^{a}	40.40 ± 3.20 ^{a}
Ketones	YC	0.31 ± 0.01 ^{c}	1.35 ± 0.14 ^{a}	1.31 ± 0.11 ^{a}	0.67 ± 0.01 ^{b}	0.23 ± 0.02 ^{cd}	0.15 ± 0.05 ^{d}
	YQY	0.73 ± 0.08 ^{c}	1.20 ± 0.02 ^{b}	1.81 ± 0.16 ^{a}	0.48 ± 0.02 ^{d}	0.12 ± 0.03 ^{e}	0.11 ± 0.00 ^{e}
	QTX	0.34 ± 0.05 ^{c}	1.08 ± 0.08 ^{b}	1.60 ± 0.03 ^{a}	0.15 ± 0.02 ^{d}	0.12 ± 0.02 ^{d}	0.09 ± 0.00 ^{d}
Fatty aldehydes	YC	1.11 ± 0.32 ^{b}	2.68 ± 0.87 ^{a}	0.23 ± 0.02 ^{c}	0.03 ± 0.00 ^{c}	0.02 ± 0.00 ^{c}	0.07 ± 0.01 ^{c}
	YQY	0.67 ± 0.01 ^{a}	0.47 ± 0.02 ^{b}	0.17 ± 0.01 ^{c}	0.01 ± 0.00 ^{e}	0.03 ± 0.01 ^{e}	0.12 ± 0.03 ^{d}
	QTX	2.19 ± 0.95 ^{a}	1.36 ± 0.21 ^{b}	0.90 ± 0.08 ^{b}	0.02 ± 0.00 ^{c}	0.03 ± 0.00 ^{c}	0.06 ± 0.01 ^{c}
Aromatic aldehydes	YC	0.06 ± 0.00 ^{e}	0.20 ± 0.02 ^{a}	0.11 ± 0.01 ^{cd}	0.13 ± 0.01 ^{bc}	0.14 ± 0.02 ^{b}	0.09 ± 0.01 ^{d}
	YQY	0.10 ± 0.01 ^{bc}	0.27 ± 0.06 ^{a}	0.31 ± 0.05 ^{a}	0.11 ± 0.03 ^{b}	0.06 ± 0.01 ^{bc}	0.04 ± 0.00 ^{c}
	QTX	0.08 ± 0.00 ^{b}	0.08 ± 0.01 ^{b}	0.20 ± 0.01 ^{a}	0.08 ± 0.01 ^{b}	0.04 ± 0.00 ^{c}	0.04 ± 0.00 ^{c}
Fatty acids	YC	0.67 ± 0.08 ^{c}	1.38 ± 0.62 ^{b}	3.89 ± 0.38 ^{a}	3.78 ± 0.32 ^{a}	1.48 ± 0.20 ^{b}	1.38 ± 0.15 ^{b}
	YQY	0.88 ± 0.24 ^{c}	3.58 ± 0.81 ^{a}	4.12 ± 0.29 ^{a}	1.81 ± 0.41 ^{b}	0.68 ± 0.19 ^{c}	1.02 ± 0.09 ^{c}
	QTX	1.30 ± 0.04 ^{b}	2.73 ± 0.62 ^{a}	2.90 ± 0.24 ^{a}	0.92 ± 0.23 ^{bc}	0.59 ± 0.05 ^{c}	0.66 ± 0.14 ^{c}
Terpenes	YC	0.06 ± 0.00 ^{b}	0.10 ± 0.01 ^{a}	0.02 ± 0.00 ^{c}	0.02 ± 0.00 ^{c}	0.01 ± 0.00 ^{c}	0.02 ± 0.00 ^{c}
	YQY	0.03 ± 0.00 ^{a}	0.03 ± 0.00 ^{a}	0.02 ± 0.00 ^{bc}	0.03 ± 0.01 ^{a}	0.01 ± 0.00 ^{c}	0.03 ± 0.00 ^{ab}
	QTX	0.16 ± 0.01 ^{a}	0.16 ± 0.01 ^{a}	0.04 ± 0.01 ^{b}	0.02 ± 0.00 ^{c}	0.02 ± 0.00 ^{c}	0.02 ± 0.00 ^{c}
Aromatic alkenes	YC	27.54 ± 3.79 ^{a}	1.20 ± 0.35 ^{d}	22.56 ± 1.16 ^{b}	10.67 ± 0.40 ^{c}	1.67 ± 0.04 ^{d}	1.10 ± 0.16 ^{d}
	YQY	27.48 ± 1.68 ^{a}	0.80 ± 0.16 ^{c}	0.87 ± 0.04 ^{c}	7.28 ± 0.38 ^{b}	1.74 ± 0.06 ^{c}	1.56 ± 0.03 ^{c}
	QTX	28.12 ± 0.88 ^{a}	1.19 ± 0.15 ^{d}	10.61 ± 1.06 ^{b}	2.96 ± 0.14 ^{c}	0.77 ± 0.05 ^{d}	0.67 ± 0.02 ^{d}
Sulfides	YC	n.d.	0.02 ± 0.00 ^{d}	0.10 ± 0.01 ^{b}	0.06 ± 0.01 ^{c}	0.13 ± 0.00 ^{a}	0.12 ± 0.01 ^{a}
	YQY	0.02 ± 0.01 ^{d}	0.15 ± 0.02 ^{a}	0.13 ± 0.02 ^{a}	0.06 ± 0.01 ^{c}	0.07 ± 0.00 ^{bc}	0.09 ± 0.00 ^{b}
	QTX	n.d.	0.01 ± 0.00 ^{cd}	0.05 ± 0.00 ^{c}	0.12 ± 0.01 ^{ab}	0.16 ± 0.01 ^{a}	0.11 ± 0.06 ^{b}
Volatile phenols	YC	0.47 ± 0.24 ^{c}	2.74 ± 0.52 ^{a}	1.39 ± 0.62 ^{b}	0.48 ± 0.02 ^{c}	0.23 ± 0.14 ^{c}	0.06 ± 0.01 ^{c}
	YQY	0.29 ± 0.08 ^{c}	0.64 ± 0.11 ^{b}	0.81 ± 0.09 ^{a}	0.17 ± 0.01 ^{cd}	0.07 ± 0.04 ^{d}	0.08 ± 0.02 ^{d}
	QTX	0.43 ± 0.29 ^{bc}	0.79 ± 0.18 ^{a}	0.62 ± 0.22 ^{ab}	0.09 ± 0.01 ^{d}	0.14 ± 0.02 ^{cd}	0.06 ± 0.03 ^{d}

Note: Different Latin letters indicate significant differences according to Duncan's test ($p < 0.05$). YC, Yinchuan sub-region; YQY, Yuquanying sub-region; QTX, Qingtongxia sub-region.

Table S4. Relative abundances of exclusive genera of three sub-regions

Sub-regions	Exclusive genera to total (%)
YC	0.41
YQY	1.84

QTX	0.19

Note: The exclusive genera in YC, YQY, and QTX were 41 (198 tags), 294 (881 tags), and 57 (91 tags), respectively. Totally 47,880 tags were obtained for each sub-region after quality control of the raw data. YC, Yinchuan sub-region; YQY, Yuquanying sub-region; QTX, Qingtongxia sub-region.

Table S5. Nonlinear regression between bacteria and VOCs

**Regression**	**VOCs**	**YC**			**YQY**			**QTX**		
		Pan.	***Rho.***	***Kom.***	***Pan.***	***Rho.***	***Kom.***	***Pan.***	***Rho.***	***Kom.***
Logistic	AE	↓	↓ *	-	↑	↑	↓	↑ *	↓	↑
	ME	↓ *	↑	-	↑ *	↓	↓	↑ *	↑	-
	IBE	↓ *	↑	-	↑ *	↑	↓	↑ *	↑	-
	IAE	↑ *	↑ *	-	↑	↑	↓	↑ *	↑	-
	SCFA	↑ *	↑	-	↑	↓	↑	↑	↓	-
	BCFA	↓ *	↓ *	-	↑ *	↑	↓	↑ *	↑	-
	AA	↓ *	↓	-	↑	↓	↓	↑ *	-	-
	FAL	↑ *	↑ *	-	↓ *	↓	↑	↓	↓	-
	AAL	↓ *	↑ *	-	↑	↑	↑	↑	↓	-
	FA	↓	↓ *	-	↑	↓	↓	↑ *	↓ *	-
	T	↑	↑ *	↓	↓ *	↓	↑	↓ *	↓	-
	AALK	↑	↓	-	↓	↑	↑	↓ *	↓	-
	S	↑	↓	-	↑	↓	↓	↑ *	-	-
Hill	AE	-	-	-	↑	-	-	↑ *	↑	-
	ME	-	-	-	↑ *	-	-	↑ *	↑ *	-
	IBE	-	↑	-	↑ *	↑	-	↑ *	↑	-
	IAE	-	-	-	↑	↑	-	↑ *	↑	-
	SCFA	↑ *	↑	-	↑	-	↑	↑	↑	-
	BCFA	-	-	-	↑ *	-	-	↑ *	↑ *	-
	AA	-	-	-	↑	-	-	↑ *	↑	-
	FAL	↑ *	↑	-	↓ *	↑ *	↑ *	-	↑	-
	AAL	↑ *	↑	-	↑	↑ *	↑ *	↑	↑	-
	FA	↑ *	-	-	↑	-	↑ *	↑ *	↑	-
	T	↑ *	↑	-	↓ *	-	↑ *	↑	↑	-
	AALK	-	-	-	↓	↑	↑	-	↑	-
	S	-	-	-	↑	-	-	↑ *	↑	-

Note: AE: acetic esters; ME: methyl esters; IBE: isobutyl esters; IAE: isoamyl esters; SCFA: straight-chain fatty alcohols; BCFA: branched-chain fatty alcohols; AA: aromatic alcohols; FAL: fatty aldehydes; AAL: aromatic aldehydes; FA: fatty acids; T: terpenes; AALK: aromatic alkenes; S: sulfides. *Pan.*, *Pantoea*; *Rho.*, *Rhodococcus*; *Kom.*, *Komagataeibacter*. A ' ↑ ' or a ' ↓ ' indicates an integral increase or decrease trend of the scatter plot between each VOC and each bacterial genus. A '-' indicates a failed nonlinear fitting. Significance of nonlinear fitting: * $p < 0.05$. YC, Yinchuan sub-region; YQY, Yuquanying sub-region; QTX, Qingtongxia sub-region.

Table S6. Nonlinear regression between bacteria and ethanol

Regression	YC			YQY			QTX		
	Pan.	***Rho.***	***Kom.***	***Pan.***	***Rho.***	***Kom.***	***Pan.***	***Rho.***	***Kom.***
Logistic	↓ *	-	↑	↑	-	↓	↑ *	↑ *	↑
Hill	-	-	-	↑ *	-	-	↑ *	↑ *	↑

Note: *Pan., Pantoea*; *Rho., Rhodococcus*; *Kom., Komagataeibacter*. A ' ↑ ' or a ' ↓ ' indicates an integral increase or decrease trend of the scatter plot between ethanol and each bacterial genus. A '-' indicates a failed nonlinear fitting. Significance of nonlinear fitting: * $p < 0.05$. YC, Yinchuan sub-region; YQY, Yuquanying sub-region; QTX, Qingtongxia sub-region.
